# Assessment of tibial rotation and meniscal movement using kinematic magnetic resonance imaging

**DOI:** 10.1186/s13018-014-0065-8

**Published:** 2014-08-21

**Authors:** Hai-Nan Chen, Kan Yang, Qi-Rong Dong, Yi Wang

**Affiliations:** 1 Department of Orthopaedics, Second Affiliated Hospital of Soochow University, Suzhou 215004, China; 2 Department of Orthopaedics, the Seventh People’s Hospital of Suzhou, Suzhou 215151, China

**Keywords:** Tibial rotation, Meniscal movement, Magnetic resonance imaging

## Abstract

**Objective:**

This work aimed to assess tibial rotations, meniscal movements, and morphological changes during knee flexion and extension using kinematic magnetic resonance imaging (MRI).

**Methods:**

Thirty volunteers with healthy knees were examined using kinematic MRI. The knees were imaged in the transverse plane with flexion and extension angles from 0° to 40° and 40° to 0°, respectively. The tibial interior and exterior rotation angles were measured, and the meniscal movement range, height change, and side movements were detected.

**Results:**

The tibia rotated internally (11.55° ± 3.20°) during knee flexion and rotated externally (11.40° ± 3.0°) during knee extension. No significant differences were observed between the internal and external tibial rotation angles (*P* > 0.05), between males and females (*P* > 0.05), or between the left and right knee joints (*P* > 0.05). The tibial rotation angle with a flexion angle of 0° to 24° differed significantly from that with a flexion angle of 24° to 40° (*P* < 0.01). With knee flexion, the medial and lateral menisci moved backward and the height of the meniscus increased. The movement range was greater in the anterior horn than in the posterior horn and greater in the lateral meniscus than in the medial meniscus (*P* < 0.01). During backward movements of the menisci, the distance between the anterior and posterior horns decreased, with the decrease more apparent in the lateral meniscus (*P* < 0.01). The side movements of the medial and lateral menisci were not obvious, and a smaller movement range was found than that of the forward and backward movements.

**Conclusion:**

Knee flexion and extension facilitated internal and external tibial rotations, which may be related to the ligament and joint capsule structure and femoral condyle geometry.

## Introduction

The knee joint, a weight-bearing structure with complicated movement patterns, is composed of the patellofemoral joint and the medial and lateral tibiofemoral joints. The functions of the knee joint include flexion, extension, adduction, abduction, and rotation, which is the most complex [1],[2]. The knee joint can maintain stability even when bearing a load 5 to 10 times its own weight. The meniscus, which is attached to the tibial plateau, has an important role in knee joint function. The meniscus can increase the contact area between the femur and the tibia to effectively distribute the load on a wider joint surface. Thus, damage caused by stress concentration on the joint cartilage is avoided. In addition, the meniscus can transfer stress and adsorb concussion while bearing a load, increase knee joint stability by deepening the tibial plateau, lubricate the joint, and nourish the cartilage [3],[4]. These functions are exhibited during knee joint activities.

Numerous studies are focused on investigating the three-dimensional motion of the knee joint by measuring tibial rotation and meniscal movement, which has certain advantages and disadvantages [5],[6]. In the present study, kinematic magnetic resonance imaging (MRI) was used to study tibial rotation, meniscal movement, and morphological changes. Tibial rotation and meniscal movement as well as their clinical significance are discussed.

## Materials and methods

### General data

Thirty healthy volunteers with ages ranging from 25 to 66 years with an average age of 41.2 years were enrolled in this study. A total of 30 knees (left knee, seven males and eight females; right knee, eight males and seven females) were examined. The inclusion criteria were as follows: (1) no history of knee injury; (2) no history of knee surgery; and (3) no history of knee disease or existing knee disease. This study was conducted in accordance with the declaration of Helsinki. This study was conducted with approval from the Ethics Committee of Second Affiliated Hospital of Soochow University. Written informed consent was obtained from all participants.

### Inspection methods

An Artoscan M dedicated-extremity MRI system (0.2 T, Esaote Company, Genoa, Italy) was used to measure tibial rotation and meniscal movement. The imaging parameters were as follows: T1 weighting, spin echo, transverse section imaging, time repetition (TR) of 230 ms, echo time of 24 ms, layer thickness of 5 mm, field of view of 20 cm × 20 cm with an average frequency of 1 for the signal, matrix of 192 × 192, signal obtaining time of 28 s, and a total inspection time of 15 min.

Volunteers were asked to lay supine. The foot on the inspection side was fixed on a lockable mobile device, and the knee was placed in a soft rectangular coil. Flexion and extension of the knee were conducted to obtain different knee joint positions. The knee joint was flexed from the extended position with transverse section imaging conducted at 0°, 8°, 16°, 24°, 32°, and 40° angle positions. Then, the knee joint was extended from the 40° angle position, and transverse section imaging was conducted at 40°, 32°, 24°, 16°, 8°, and 0°. At the same time, sagittal imaging and coronal imaging were conducted on the medial and lateral menisci, respectively, to investigate the meniscal movement and morphological changes during knee flexion. Six physicians, from the three groups (with one physician from the imaging department and one from each clinical department, in each group) participated in the MRI reading independently. There was no intergroup correlation among the doctors.

## Results

**Figure 1:**
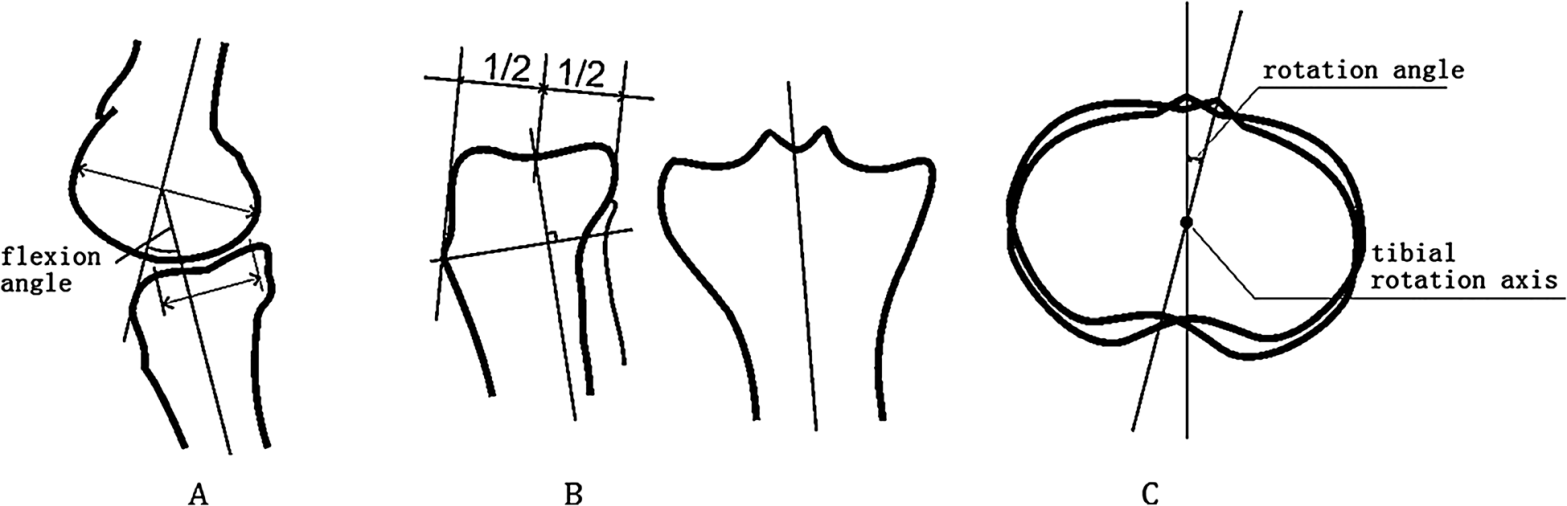
**The angle of the tibial rotation. (A)** Tibial flexion angle; **(B)** tibial rotation axis; **(C)** tibial rotation angle.

According to the measurement standard described by Sanfridsson et al. [7], the knee flexion angle was defined as the angle between the femoral longitudinal axis and the tibial longitudinal axis. The tibial rotation axis was defined as the vertical line from the midpoint of the anterior and posterior diameters in the tibial eminence, parallel to the tibial posterior edge. The tibial rotation angle was defined as the rotation angle of the sagittal plane through the rotation axis. In this study, layered transverse section imaging parallel to the joint surface was performed from the tibial plateau joint cartilage to the tibial tubercle plane. According to geometric principles, the tibial rotation angle was defined as the rotation angle through the line from the tibial tubercle midpoint to the upper tibiofibular joint midpoint, an accurate and convenient measurement. Overlapping images were obtained to measure the angle of the tibial rotation (Tables 1, 2, 3 and Figure 1).

**Table 1:** Relationship between tibial rotation and knee flexion angle

**Flexion angle**	**0°–40°**	**0°–8°**	**−16°**	**−24°**	**−32°**	**−40°**
Tibial external rotation	11.40**°** ± 3.00°*	3.10**°** ± 0.85**°**	3.10**°** ± 0.91**°**	2.35**°** ± 1.04**°**	1.45**°** ± 0.60**°**	1.35**°** ± 0.49**°**
Tibial internal rotation	11.55**°** ± 3.20**°**	2.95**°** ± 1.05**°**	2.65**°** ± 0.93**°**	2.45**°** ± 1.00**°**	1.95**°** ± 0.83**°**	1.55**°** ± 0.60**°**

*P > 0.05 compared with tibial external rotation.

**Table 2:** Relationship between tibial rotation and gender

**Gender**	**Tibial external rotation**	**Tibial internal rotation**
Male	11.22**°** ± 2.54**°**	11.33**°** ± 3.32**°**
Female	11.55**°** ± 3.45**°**	11.73**°** ± 3.26**°**
*P*	>0.05	>0.05

**Table 3:** Relationship between tibial rotation and knee position

**Knee position**	**Tibial external rotation**	**Tibial internal rotation**
Left knee	11.18**°** ± 2.75**°**	11.00**°** ± 2.93**°**
Right knee	11.67**°** ± 3.43**°**	12.22**°** ± 3.56**°**
*P*	>0.05	>0.05

**Figure 2:**
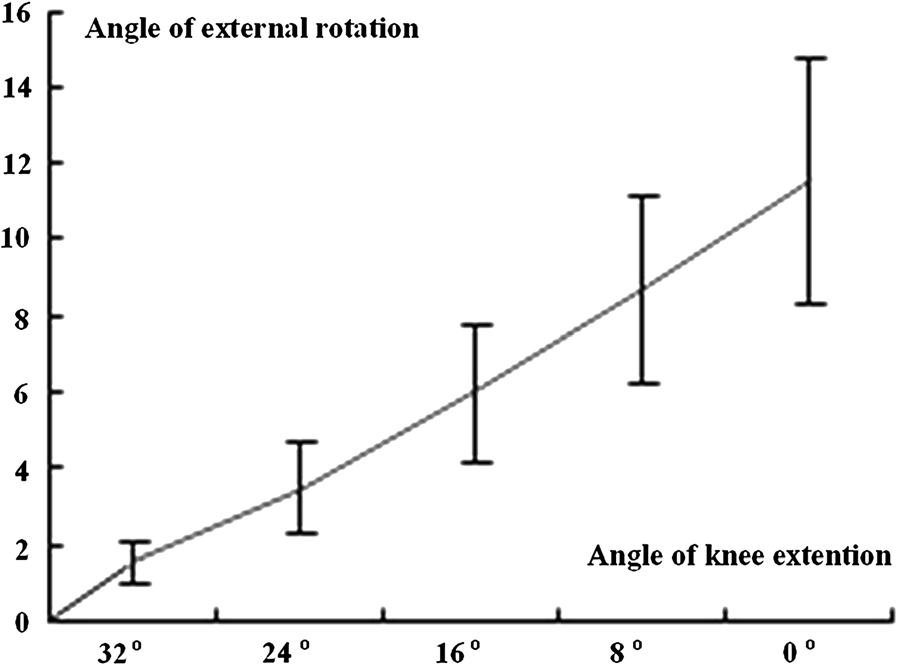
Relationship between tibial external rotation and knee extention.

**Figure 3:**
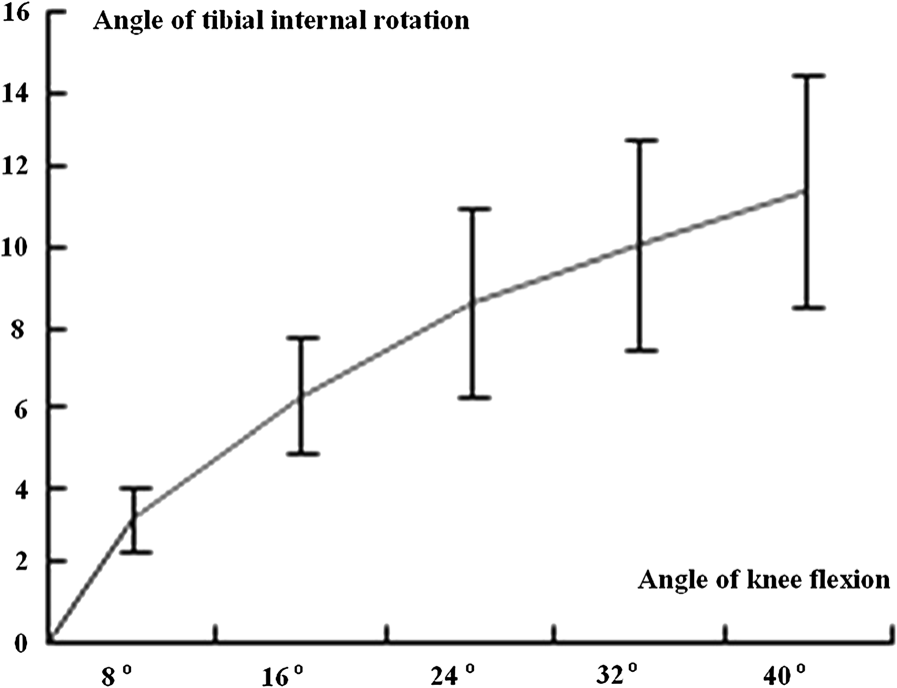
Relationship between tibial internal rotation and knee flexion.

The flexion and extension process was divided into five stages, the tibial rotation angle was recorded, and statistical analysis was conducted (Tables 4 and 5). The results revealed no significant difference between the internal and external tibial rotation angles (*P* > 0.05), between males and females (*P* > 0.05), and between the left and right knee joints (*P* > 0.05). The tibial rotation angle with knee flexion angles between 0° and 24° was significantly different from the rotation angle with flexion angles between 24° and 40° (*P* < 0.01). This result indicated that tibial rotation at knee flexion angles of 0° to 24° is stable and regular, whereas obvious and irregular changes were observed for flexion angles exceeding 24°. The tibial rotation angle gradually decreased with an increasing knee flexion angle. Figures 2 and 3 show that early tibial external and later tibial internal rotations were not obvious, but later internal rotation and early external rotation were very obvious.

**Table 4:** Relationship between tibial internal rotation and knee flexion angle

**Flexion angle**	**0°–8°**	**0°–16°**	**1°–24°**	**0°–32°**	**0°–40°**
Tibial internal rotation	3.14**°** ± 0.85**°**	6.24**°** ± 1.48**°**	8.62**°** ± 2.36**°**	10.05**°** ± 2.67**°**	11.40**°** ± 3.0**°**

**Table 5:** Relationship between tibial external rotation and knee extention angle

**Extention angle**	**40°–32°**	**40°–24°**	**40°–16°**	**40°–8°**	**40°–0°**
Tibial external rotation	1.55**°** ± 0.6**°**	3.5**°** ± 1.19**°**	5.95**°** ± 1.88**°**	8.65**°** ± 2.41**°**	11.55**°** ± 3.2**°**

**Figure 4:**
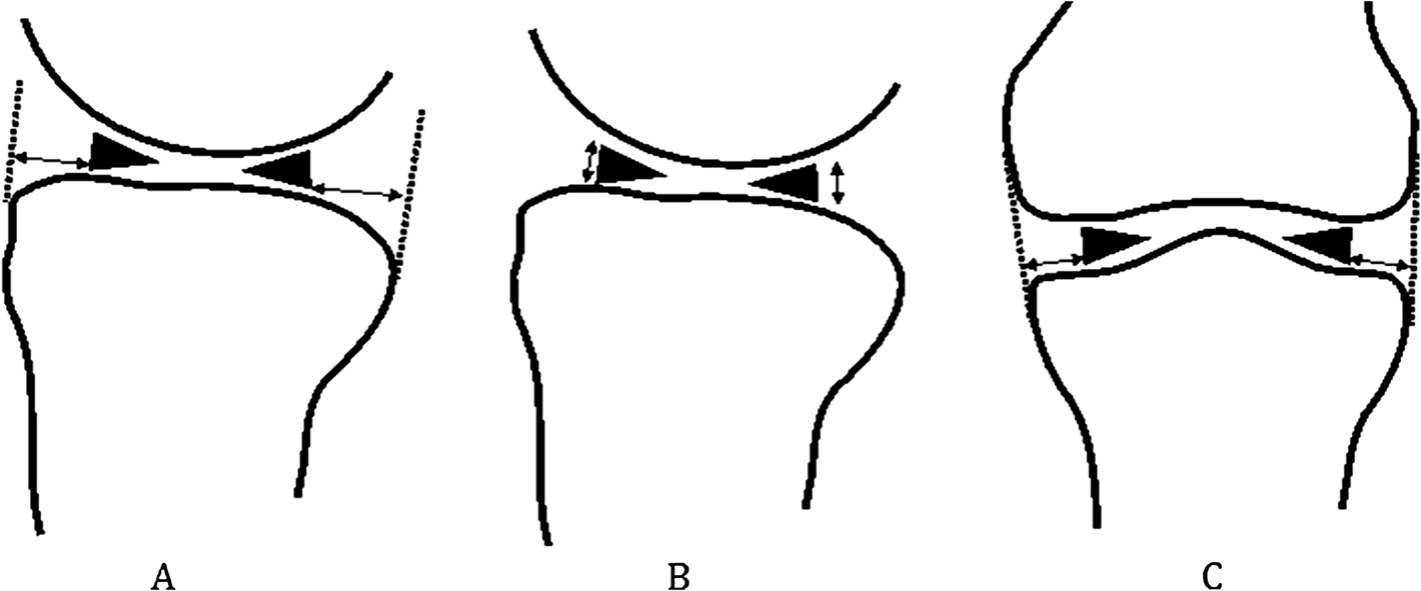
**Movements and height change of meniscus. (A)** Anteroposterior movement of meniscus; **(B)** height change of meniscus; **(C)** side movement of meniscus.

Sagittal imaging and coronal imaging were performed on the medial and lateral menisci of 30 knee joints at flexions of 0° to 40°. The movement of and morphological changes in the anterior and posterior horn in the meniscus were observed. With knee flexion, the medial and lateral menisci moved backward, and the height of the anterior and posterior horns increased to a differing extent. The medial and lateral menisci also moved inward and outward, respectively. Table 6 shows that the movement range of the anterior horn was larger than that of the posterior horn and that of the lateral meniscus was larger than that of the medial meniscus (*P* < 0.01). The distance between the anterior and posterior horns decreased with the backward movement of the meniscus. This movement was more obvious for the lateral meniscus (*P* < 0.01). The side movements of the medial and lateral menisci were not obvious, and a smaller movement range was found than that of the forward and backward movements (Figure 4).

**Table 6:** Meniscal movement and height changes during knee flexion (mm)

**Index**	**Medial meniscus**	**Lateral meniscus**
Movement of anterior horn	5.96 ± 1.88	8.98 ± 2.13
Movement of posterior horn	4.76 ± 1.40	6.68 ± 1.71
Height change of anterior horn	2.26 ± 0.51	2.92 ± 0.44
Height change of posterior horn	1.81 ± 0.49	2.28 ± 0.44
Change of anteroposterior diameter	4.23 ± 0.91	5.28 ± 1.20
Side movement	1.75 ± 0.18	2.02 ± 0.26

## Discussion

The knee joint has a special structure, a complex composition, and important functions. It is composed of the femoral condyle, tibial plateau, and patella, combined with a joint capsule, ligament, meniscus, and surrounding muscle tissue. The knee joint implements internal and external rotation through flexion, extension, adduction, and abduction. The knee joint has a screw-home mechanism [8],[9], where the joint is locked by tibial external rotation during the process of extension and is then unlocked during the process of flexion. Therefore, the knee joint is highly stable during full extension, with no rotation and side movement. The tibial intercondylar eminence can limit inward and outward knee movement and can elevate the femur during tibial rotation because the anterior and posterior cruciate and lateral collateral ligaments restrict excessive tibial rotation. The joint ligaments are tightened, which further limits tibial rotation and enhances knee joint stability. Some scholars [8],[10] believe that the vertical axis for knee joint rotation is located in the medial femoral intercondylar eminence. With increasing flexion angle, it gradually shifts rearward and closer to the posterior cruciate ligament. Tibial rotation can occur during passive knee flexion and extension. During daily activities, many muscles are used in tibial internal rotation, such as the popliteal muscle, semitendinosus, semimembranosus, sartorius, and gracilis, and in external rotation, such as the biceps femoris and vastus lateralis [10],[11]. Therefore, the knee joint is more stable during extension than flexion, but it can rotate with sideward movement during flexion to adapt to different motion states.

The meniscus is a semilunar fibrocartilage pad in the knee joint between the femur and the tibia. The wedge structure of the transverse section deepens the articular surface of the tibial plateau. Therefore, the prominent femoral and tibial condyles are well matched. Only a loose ligament exists between the lateral meniscus and the tibia and joint capsule, and a popliteal tendon exists between the lateral meniscus and fibular collateral ligament [12]. A total of 70% of the posterior horns in the lateral meniscus are connected to the medial femoral condyle through one or two meniscofemoral ligaments (the posterior meniscofemoral ligament or Wrisberg ligament, and the anterior meniscofemoral ligament or Humphrey ligament). The Wrisberg ligament is found in most knee joints, and 6% of knee joints have both kinds of ligament [12],[13]. The medial meniscus presents as a ‘C’ shape. The anterior and posterior horns are attached to the anterior and posterior areas of the tibial eminence but are distant from each other. A stronger connection exists between the medial meniscus and the tibial plateau and joint capsule. The medial meniscus is also closely attached to the tibial collateral ligament. Therefore, the flexibility of the medial meniscus movement is less than that of the lateral meniscus. The anterior horns of the medial and lateral menisci are connected by a transverse ligament, which obviously limits the rearward movement of the anterior horn. Muhle et al. [14] studied the effect of the transverse ligament on meniscal movement and found a significant difference in the meniscal movement range before and after cutting the transverse ligament. This result further confirmed the limiting effect of the transverse ligament on meniscal movement. The main function of the meniscus is to transfer the load. The meniscus must bear the thrust force toward around, leading to meniscal movement. The popliteal muscle contracts and causes a backward movement in the meniscus during knee flexion, because the hamstring tendon attachment is in the posterior horn of lateral meniscus. The meniscofemoral ligament, which is attached to the posterior horn of the lateral meniscus, can cause a backward movement of the posterior lateral meniscus during knee flexion and tibial internal rotation [15],[16]. The attachments of the medial and lateral menisci with the tibia and joint capsule as well as the annular structure in the transverse ligament can limit the excessive external movement of the meniscus [17].

Many studies were conducted on human knee joint kinematics and kinetics. However, most were cadaveric studies, lacking the *in vivo* environmental tension of muscles and ligaments. Thus, *in vivo* knee joint movement is not accurately reflected. *In vivo* research using fixing belts or skin-marked sensors is a non-invasive method, but the influences of skin and soft tissue cause large errors [5]. In Eberhart’s study, drift bolting was performed on the femur and tibia of 11 subjects, and the spectrophotometric analysis results revealed that the tibial rotation angle ranges from 4° to 13.3° with an average of 8.7° [6]. Kettelkamp used an electronic goniometer to measure the tibial rotation angle and found that the tibial rotation range was from 5.7° to 25.3° with averages of 12.9° (right knee) and 13.3° (left knee). Tibial rotation during walking was also studied. The results showed that maximum extension and external rotation occur before heel touchdown. Flexion and internal rotation occur before heel touchdown and continue until an absolute standing position is reached [7]. In Nilsson’s study, 0.8-mm tantalum beads were implanted in the tibia and femur, and radiographic stereo photogrammetry was conducted on tibial rotation [5]. These methods improve measurement accuracy but are invasive and difficult to apply clinically.

In this study, the internal and external rotation of the tibia during knee flexion and extension were measured using magnetic resonance technology. The results revealed that the tibia internally rotates (11.55° ± 3.20°) during knee flexion and externally rotates (11.40° ± 3.0°) during knee extension, which are the same as the results obtained by Ahrens et al. [6]. No significant statistical differences in the rotation angle between males and females and between the left and right knee joints were observed because the small difference between the internal and external rotations cannot be measured. The tibial rotation with a knee flexion angle range of 0° to 24° was also stable and regular with obvious and irregular changes for flexion angles exceeding 24°.

MRI was used for transverse section scanning on the upper tibia rotation. MRI is not affected by the surrounding muscles, ligaments, or soft tissues and is highly accurate and non-invasive. Therefore, MRI provides a useful index for clinically evaluating knee joint diseases. If tibial rotation can be measured during routine examination, the surgical process can be simplified and useful clinical data can be obtained.

The meniscus has an important function in knee extension and flexion. Previous studies on meniscal movement were performed on corpses, which required joint incision. These studies were unable to reflect the actual situation of the meniscus because of poor accuracy [18]. MRI can clearly show the condition of the meniscus because of good tissue resolution. In addition, MRI is suitable for studying the movement and morphologic changes of the meniscus. The results of this study show that the medial and lateral menisci move backward with knee flexion, and the movement range of the lateral meniscus is larger than that of the medial meniscus (*P* < 0.01). The movement range of the anterior horn is also larger than that of the posterior horn, while the posterior horn of the medial meniscus has the smallest range. This result may be caused by the relationship of meniscal movement with femoral condyle shape and motion. The convex femoral condyle slides and rolls on the tibial plateau with knee flexion and inevitably pushes the meniscus to move backward. The meniscus is gradually pushed to the side during flexion to match the meniscus with the femoral condyle and tibial plateau as far as possible because the posterior width of the femoral condyle is greater than that of the anterior femoral condyle [12]. The meniscus is always embedded between the femoral condyle and the tibial condyle, which increases the stability of the knee joint and prevents femur movement and the embedding of the synovium and joint capsule. During the process of flexion, the meniscus moves backward and the anteroposterior diameter gradually decreases. This result may be related to the shape and position of the femoral condyle. The tibiofemoral contact area gradually decreases during flexion because of the large curvature radius at the femoral condyle top and the reduced rearward radius. Therefore, the load can be uniformly transferred, and damage to the meniscus can be prevented. The movement range of the lateral meniscus was also greater than that of the medial meniscus, and the movement range of the anterior horn was larger than that of the posterior horn. The smallest movement range was found in the medial meniscus; it is immovable and vulnerable to injury. This finding is consistent with the results of previous studies [19],[20].

Tibial rotation is influenced by the knee joint bone morphogenetic structure combined with ligaments. The tibial collateral ligament, anterior and posterior cruciate ligaments, and joint capsule are involved in tibial rotation and control excessive rotation. Therefore, excessive tibial rotation caused by trauma can damage the bone morphogenetic structure and lead to rotation instability. The rupture of the tibial collateral ligament can cause a significant increase in tibial external rotation but has little effect on tibial internal rotation [21]. However, anterior cruciate ligament rupture can lead to an increase in tibial internal rotation, and posterior cruciate ligament rupture can cause excessive external rotation. Ruptures in both the anterior and posterior cruciate ligaments can significantly increase tibial internal and external rotation. Therefore, the tibial rotation angle can be used as an important metric in evaluating knee ligament injury. Stress distribution on the patellofemoral joint is affected by tibial rotation. Excessive internal or external rotation can lead to excessive load on the medial and lateral patellofemoral joint surface with insufficient stress stimulation on either side. This can cause a series of injuries to the joint cartilage and subchondral tissue and could aggravate patellofemoral joint degeneration [22].

In this study, the height of the meniscus was also studied. The results showed that meniscus height gradually increased with knee flexion. The meniscus undergoes morphological changes to adapt to the smaller curvature radius of the posterior femoral condyle. The meniscal matrix also contains a large number of type I collagen and proteogly can, both of which have a strong absorption ability and the capability to enhance the resistance compression of tissues and the elasticity of the meniscus [23]. The hardness of the meniscus is half that of the cartilage under compression. Therefore, the meniscus has a strong ability to disperse stress and fully absorb the concussion [23],[24]. The morphological changes of the meniscus may be related to nutrient absorption. With knee joint movement, the meniscus can present forward, backward, and sideward movements with morphological changes to adapt to the load and exert important functions. Meniscus injury or resection has adverse effects on the knee joint. Injury or resection can significantly change the load-transferring mode of the knee joint and cause an overload on the joint surface and subsequent degeneration of the joint cartilage. Kim et al. [25] found that the severity of these changes is related to the resection amount of the meniscus tissue. When treating an injury, the peripheral portions of the meniscus should be retained as much as possible and should be combined with meniscus repair, allografting, and prosthetic replacement. This method can aid in full recovery of the meniscus function.

## Conclusion

Normally, as the knee extends and flexes, the tibia rotates externally and internally. At this time, a screw-home mechanism occurs. This increases the stability of the knee. Rotatory stability of the knee joint is provided primarily by the ligamentous and capsular structures and by the geometry of the condyles, with muscle activity also playing a role. From our research, we know that as the knee flexes, the menisci move posteriorly and change their shape, which may be related to the appearance of femoral condyles as well as the ligament and knee joint capsule.

## Competing interests

The authors declare that they have no competing interests.

## Authors’ contributions

HC and KY are the designers of the research topic and chief persons in charge of the project, quality controloperation, data collection, data process, and article writing. QD is the director of the research topic design, coordinating the works of all sections and is in charge of quality control and article checking and modification. YW is the person performing the detailed operation and quality control. All authors read and approved the final manuscript.

## Authors’ information

Hainan Chen and Kan Yang are the co-first authors.

## References

[B1] Wang P Zhao Z Fu W Xu H Advancement of rotational alignment of femoral prosthesis in total knee arthroplasty Zhongguo Xiu Fu Chong Jian Wai Ke Za Zhi 2011 25 1140 1144 21991827

[B2] Testa R Chouteau J Viste A Cheze L Fessy MH Moyen B Reproducibility of an optical measurement system for the clinical evaluation of active knee rotation in weight-bearing, healthy subjects Orthop Traumatol Surg Res 2012 98 159 166 10.1016/j.otsr.2011.08.017 22336486

[B3] Wyss JF Foye PM Stitik TP An infected, extruded lateral meniscal cyst as a cause of knee symptoms Am J Phys Med Rehabil 2010 89 175 176 10.1097/PHM.0b013e3181ca2431 20068436

[B4] Mastrokalos DS Papagelopoulos PJ Mavrogenis AF Hantes ME Paessler HH Changes of the posterior meniscal horn height during loading: an in vivo magnetic resonance imaging study Orthopedics 2008 31 68 10.3928/01477447-20080101-28 19292170

[B5] Ishii Y Terajima K Terashima S Koga Y Three-dimensional kinematics of the human knee with intracorticd pin fixation Clin Orthop 1997 343 144 150 9345219

[B6] Ahrens P Kirchhoff C Fischer F Heinrich P Eisenhart-Rothe R Hinterwimmer S Kirchhoff S Imhoff AB Lorenz SG A novel tool for objective assessment of femorotibial rotation: a cadaver study Int Orthop 2011 35 1611 1620 10.1007/s00264-010-1159-5 21181404 PMC3193962

[B7] Sanfridsson J Ryd L Svahn G Fridén T Jonsson K Radiographic measurement of femorotibial rotation in weight-bearing Acta Radiol 2011 42 202 217 10.1080/028418501127346512 11259950

[B8] Amiri S Cooke D Kim IY Wyss U Mechanics of the passive knee joint. Part 2: interaction between the ligaments and the articular surfaces in guiding the joint motion Proc Inst Mech Eng H 2007 221 821 832 10.1243/09544119JEIM181 18161242

[B9] Keays SL Sayers M Mellifont DB Richardson C Tibial displacement and rotation during seated knee extension and wall squatting: a comparative study of tibiofemoral kinematics between chronic unilateral anterior cruciate ligament deficient and healthy knees Knee 2013 20 346 353 10.1016/j.knee.2012.07.005 22854170

[B10] Lee TQ Morris G Csintalan RP The influence of tibial and femoral rotation on patellofemoral contact area and pressure J Orthop Sports Phys Ther 2003 33 686 693 10.2519/jospt.2003.33.11.686 14669964

[B11] Hoshino Y Araujo P Ahlden M Moore CG Kuroda R Zaffagnini S Karlsson J Fu FH Musahl V Standardized pivot shift test improves measurement accuracy Knee Surg Sports Traumatol Arthrosc 2012 20 732 736 10.1007/s00167-011-1850-0 22205096

[B12] Kawahara Y Uetani M Fuchi K Eguchi H Hayashi K MR assessment of movement and morphologic change in the menisci during knee flexion Acta Radiol 1999 40 610 614 10.3109/02841859909175596 10598848

[B13] Kim JE Choi SH Is the location of the Wrisberg ligament related to frequent complete discoid lateral meniscus tear? Acta Radiol 2010 51 1120 1125 10.3109/02841851.2010.520026 21062131

[B14] Muhle C Thompson WO Sciulli R Pedowitz R Ahn JM Yeh L Clopton P Haghighi P Trudell DJ Resnick D Transverse ligament and its effect on meniscal motion correlation of kinematic MR imaging and anatomic sections Invest Radiol 1999 34 558 565 10.1097/00004424-199909000-00002 10485070

[B15] d’Entremont AG Wilson DR Joint mechanics measurement using magnetic resonance imaging Top Magn Reson Imaging 2010 21 325 334 10.1097/RMR.0b013e31823fb2b9 22129645

[B16] Park JS Ryu KN Yoon KH Meniscal flounce on knee MRI: correlation with meniscal locations after positional changes AJR Am J Roentgenol 2006 187 364 370 10.2214/AJR.05.0339 16861539

[B17] Tibesku CO Mastrokalos DS Jagodzinski M Pässler HH MRI evaluation of meniscal movement and deformation in vivo under load bearing condition Sportverletz Sportschaden 2004 18 68 75 10.1055/s-2004-813001 15164291

[B18] Vedi V Williams A Tennant SJ Spouse E Hunt DM Gedroyc WM Meniscal movement: an in-vivo study using dynamic MRI J Bone Joint Surg 1999 81 37 41 10.1302/0301-620X.81B1.8928 10067999

[B19] Bylski-Austrow DI Ciarelli MJ Kayner DC Matthews LS Goldstein SA Displacements of the menisci under joint load: an in vitro study in human knees J Biomench 1994 27 421 431 10.1016/0021-9290(94)90018-3 8188723

[B20] Thompson WO Thaete FL Fu FH Dye SF Tibial meniscal dynamics using three-dimensional reconstruction of magnetic resonance images Am J Sports Med 1991 19 210 215 10.1177/036354659101900302 1867329

[B21] Lam MH Fong DT Yung PS Chan KM Biomechanical techniques to evaluate tibial rotation. A systematic review Knee Surg Sports Traumatol Arthrosc 2012 20 1720 1729 10.1007/s00167-011-1665-z 21912885

[B22] Digby CJ Lake MJ Lees A High-speed non-invasive measurement of tibial rotation during the impact phase of running Ergonomics 2005 48 1623 1637 10.1080/00140130500101304 16338728

[B23] Fithian DL Kelly MA Mow VC Material properties and structure-function relationships in the menisci Clin Ortlop 1990 252 19 31 2406069

[B24] Szomor ZL Martin TE Bonar F Murrell GA The protective effects of meniscal transplantation on cartilage J Bone Joint Surg 2001 82 80 88 10653087 10.2106/00004623-200001000-00010

[B25] Kim JG Lee YS Bae TS Ha JK Lee DH Kim YJ Ra HJ Tibiofemoral contact mechanics following posterior root of medial meniscus tear, repair, meniscectomy, and allograft transplantation Knee Surg Sports Traumatol Arthrosc 2013 21 2121 2125 10.1007/s00167-012-2182-4 22955146

